# Clinical features and comorbidities of Epstein–Barr virus infection in childhood-onset systemic lupus erythematosus with a focus on macrophage activation syndrome: a cross-sectional study of 200 patients

**DOI:** 10.3389/fimmu.2026.1714490

**Published:** 2026-01-26

**Authors:** Hongye Wang, Mengqian Zhouyang, Xiran Yang, Jia Zhang, Helin Yan, Yingyi Zhang, Li Li, Bo Zhao

**Affiliations:** 1 Nephrology, Rheumatology and Immunology Department, Kunming Children’s Hospital (Children's Hospital Affiliated to Kunming Medical University), Kunming, Yunnan, China; 2 Kunming Key Laboratory of Children Infection and Immunity, Yunnan Key Laboratory of Children's Major Disease Research, Yunnan Medical Center for Pediatric Diseases, Yunnan Institute of Pediatrics, Kunming, Yunnan, China

**Keywords:** child, comorbidity, Epstein-Barr virus, macrophage activation syndrome, risk factors, systemic lupus erythematosus

## Abstract

**Objective:**

This study aimed to investigate the prevalence of Epstein–Barr virus (EBV) infection in patients with childhood-onset systemic lupus erythematosus (cSLE) in Southwest China and to explore its associations with clinical manifestations, laboratory parameters, disease activity, and complications, particularly macrophage activation syndrome (MAS).

**Methods:**

A single-center, retrospective, cross-sectional study was conducted, enrolling 200 cSLE patients newly diagnosed at the Yunnan Provincial Children’s Medical Center between January 2022 and June 2024. Based on EBV DNA levels tested at baseline (disease diagnosis) using real-time quantitative PCR (viral load ≥ 500 copies/mL defined as positive), patients were categorized into an EBV-positive group (*n* = 65) and an EBV-negative group (*n* = 135). Demographic data, clinical manifestations, laboratory findings, disease activity (SLEDAI-2K score), and complications [MAS, lupus nephritis (LN), neuropsychiatric SLE (NPSLE), lupus pneumonitis] were compared between the two groups. Binary logistic regression analysis was used to identify independent risk factors for MAS.

**Results:**

The prevalence of EBV infection was 32.5% (65/200). Compared to the EBV-negative group, the EBV-positive group had significantly higher frequencies of neurological symptoms (66.2% vs. 45.2%, *P* = 0.007) and serous cavity effusion (60.0% vs. 43.7%, *P* = 0.035). Laboratory analysis revealed significantly lower levels of complement C3 (*P* = 0.032) and C4 (*P* = 0.009), lower serum albumin (*P* = 0.025), and higher blood urea nitrogen (BUN) (*P* = 0.014) in the EBV-positive group. Most critically, the incidence of MAS was significantly higher in the EBV-positive group (15.4% vs. 6.7%, *P* = 0.049). Multivariate analysis confirmed that EBV infection was an independent risk factor for MAS (OR = 2.90, 95% CI: 1.01–8.69, *P* = 0.0497), while a higher platelet count was a protective factor (OR = 0.25, 95% CI: 0.07–0.75, *P* = 0.0218). No significant differences were found between the groups regarding the prevalence of LN, NPSLE, lupus pneumonitis, or the overall SLEDAI-2K score.

**Conclusion:**

EBV infection is independently associated with an increased risk of MAS in cSLE patients and is linked to more pronounced complement consumption and specific clinical manifestations such as neurological symptoms and serositis. This study underscores the importance of EBV screening in cSLE patients for the early vigilance and management of MAS.

## Introduction

Systemic lupus erythematosus (SLE) is a complex autoimmune disease characterized by multi-organ involvement and diverse autoantibody production (1). Childhood-onset SLE (cSLE), representing 15%–20% of all cases, typically follows a more aggressive disease course than adult-onset SLE, with accelerated organ damage and substantial morbidity (2). The etiology of cSLE involves intricate interactions between genetic susceptibility and environmental triggers (3).

Among environmental factors, Epstein–Barr virus (EBV) has long been implicated in SLE pathogenesis (4). While serological evidence of past EBV infection is nearly universal in adults, studies suggest that patients with SLE exhibit altered EBV serology and impaired control of latent infection (5). Proposed mechanisms include molecular mimicry, chronic B-cell activation, and apoptosis interference (6). However, despite extensive research in adult SLE, studies focusing specifically on cSLE populations remain limited and have yielded inconsistent results regarding EBV’s association with clinical manifestations and disease severity.

This gap is particularly relevant concerning macrophage activation syndrome (MAS), a life-threatening complication of cSLE characterized by excessive immune activation and cytokine storm (7). Although EBV is a recognized trigger of hemophagocytic lymphohistiocytosis, robust evidence linking EBV infection to MAS risk in cSLE is scarce, with current understanding relying mainly on case reports (8, 9).

Given the clinical heterogeneity of cSLE and the high mortality associated with MAS, identifying specific risk factors is crucial for improving outcomes. We therefore conducted this cross-sectional study to investigate the prevalence of active EBV infection, defined by DNA viremia, in a well-characterized cSLE cohort from Southwest China. We specifically aimed to analyze its associations with distinctive clinical features, laboratory parameters, and complications, with particular emphasis on MAS. Our findings aim to provide evidence for early risk stratification and optimized management in this vulnerable population.

## Materials and methods

### Study design

This was a single-center, retrospective, cross-sectional study approved by the Ethics Committee of Kunming Children’s Hospital (Approval No.: 2024-03-003-K01). The requirement for informed consent was waived as the study utilized only anonymized retrospective data. The study enrolled 200 children with childhood-onset systemic lupus erythematosus diagnosed at our hospital between January 2022 and June 2024. All patients strictly met the 2019 European Alliance of Associations for Rheumatology/American College of Rheumatology (EULAR/ACR) international classification criteria for SLE, and diagnoses were confirmed by at least two senior pediatric rheumatologists. A critical feature of our study design is that all laboratory investigations and clinical assessments were performed at the time of initial cSLE diagnosis, prior to the initiation of any systemic immunosuppressive therapy. This approach ensures that the observed associations reflect the intrinsic disease pathophysiology unaffected by treatment confounders.

### Study population

Patients were categorized into groups based on peripheral blood EBV DNA load results. Detection was performed using real-time quantitative PCR, with a viral load ≥500 copies/mL defined as positive for EBV infection. Accordingly, patients were divided into an EBV-positive group (*n* = 65) and an EBV-negative group (*n* = 135). To facilitate in-depth analysis of complications, diagnostic criteria for four major disease subtypes were explicitly defined:

Lupus nephritis: 1) Diagnosis was based on the 2024 revised pathological classification criteria of the International Society of Nephrology/Renal Pathology Society (ISN/RPS).

Macrophage activation syndrome: 2) Diagnosis required fulfillment of at least 5 of the following 8 criteria: 1) fever (>38.5°C for >7 days); 2) splenomegaly; 3) cytopenias (affecting ≥2 of 3 lineages); 4) hypertriglyceridemia (fasting triglycerides > 3 mmol/L) and/or hypofibrinogenemia (fibrinogen <1.5 g/L); 5) hemophagocytosis in the bone marrow, spleen, or lymph node; 6) low or absent NK-cell activity; 7) ferritin ≥500 μg/L; and 8) elevated soluble CD25.

Neuropsychiatric: 3) Diagnosis was based on the 1999 American College of Rheumatology (ACR) nomenclature and case definitions, combined with neuroimaging and cerebrospinal fluid analysis for comprehensive assessment.

Lupus pneumonitis: 4) Diagnosis adhered to the standards set forth in the 2020 Chinese guidelines for the diagnosis and treatment of SLE. This required a confirmed SLE diagnosis alongside respiratory symptoms (e.g., cough, dyspnea, chest pain) and new pulmonary infiltrates (e.g., ground-glass opacities, consolidation, or interstitial changes on chest CT), after excluding other causes such as pulmonary infection, heart failure, or uremic lung.

### Data collection

Data collected and analyzed retrospectively included demographic baseline information, clinical manifestations, laboratory parameters, disease activity, and treatment complications. Specifically, baseline information such as sex, age at diagnosis, and disease duration was recorded. Documented clinical manifestations included fever, characteristic rash, oral ulcers, alopecia, joint swelling/tenderness, serous cavity effusion, and neurological symptoms. Laboratory parameters comprised complete blood count, urinalysis, 24-h urine protein quantification, erythrocyte sedimentation rate (ESR), C-reactive protein (CRP), autoantibody profiles (including ANA, anti-dsDNA, anti-Sm, etc.), complement C3 and C4, and immunoglobulins, as well as EBV-specific serological antibodies and DNA load. Disease activity was quantified using the Systemic Lupus Erythematosus Disease Activity Index 2000 (SLEDAI-2K) and graded accordingly.

All data were extracted from the Hospital Information System (HIS), Laboratory Information System (LIS), and Picture Archiving and Communication System (PACS) of Kunming Children’s Hospital. To ensure accuracy and consistency, two trained researchers independently collected data using predesigned standardized forms. After the collection, a third senior researcher performed cross-verification. Any discrepancies were resolved by reviewing the original medical records for final adjudication. All data were anonymized upon entry into the analysis database, identified only by a study code to strictly protect patient privacy.

### Statistical analysis

Binary logistic regression was selected as the primary analytical method as it is the most appropriate and powerful tool for identifying associations between baseline exposures and a binary outcome at a defined cross-sectional time point. Variables pertaining to immunosuppressive therapy were not included in the regression models as all patients were treatment-naive at baseline, making these variables statistically non-informative for this analysis.

Statistical analyses were performed using R software (version 4.5.0) and SPSS Statistics (version 25.0). Normally distributed continuous data are presented as mean ± standard deviation (* $\bar{\text{x}}$* ± SD) and compared using the independent samples *t*-test. Non-normally distributed continuous data are presented as median (interquartile range) [*M* (IQR)] and compared using the Mann–Whitney *U* test. Categorical data are presented as number (percentage) [*n* (%)] and compared using the chi-square test or Fisher’s exact test, as appropriate. Variables showing statistically significant differences (*P* < 0.05) in univariate analyses were included in a binary logistic regression model for multivariate analysis to explore independent associations between EBV infection and clinical outcomes. Results are expressed as odds ratios (ORs) with 95% confidence intervals (CIs). All tests were two-sided, and a *P*-value <0.05 was considered statistically significant.

## Results

### Demographic characteristics

This study included 200 confirmed cSLE patients, stratified by EBV infection status into an EBV-positive group (*n* = 65, 32.5%) and an EBV-negative group (*n* = 135, 67.5%). The two groups demonstrated good baseline comparability in demographic characteristics. Female patients predominated in the overall cohort (*n* = 161, 80.50%) versus male patients (*n* = 39, 19.50%). The proportion of female patients was similar between the EBV-positive (*n* = 53, 81.54%) and EBV-negative groups (*n* = 108, 80.00%; *P* = 0.95). The median age at diagnosis was 11 years (range: 1–16 years) for all patients, with no significant difference between the EBV-positive (median: 11 years, range: 3–16) and EBV-negative groups (median: 11 years, range: 1–15; *P* = 0.44). Ethnically, the majority were Han (*n* = 155, 77.50%), while 45 patients (22.50%) belonged to 12 minority groups, including Dai (*n* = 10, 5.00%), Yi (*n* = 9, 4.50%), Miao (*n* = 5, 2.50%), and Hani (*n* = 5, 2.50%). Ethnic distribution did not differ significantly between groups (*P* = 0.14). All baseline demographic data are summarized in **Table 1**.

**Table 1:** Baseline demographics of cSLE patients with and without EBV infection.

Characteristics	Positive (*N* = 65)	Negative (*N* = 135)	Total (*N* = 200)	*P*-value
Sex				0.95
Female	53 (26.50%)	108 (54.00%)	161 (80.50%)	
Male	12 (6.00%)	27 (13.50%)	39 (19.50%)	
Age				0.44
Median [min–max]	11.00 [3.00, 16.00]	11.00 [1.00, 15.00]	11.00 [1.00, 16.00]	
Ethnicity				0.14
Bai	1 (0.50%)	1 (0.50%)	2 (1.00%)	
Bulang	0 (0.0e+0%)	1 (0.50%)	1 (0.50%)	
Dai	5 (2.50%)	5 (2.50%)	10 (5.00%)	
Han	47 (23.50%)	108 (54.00%)	155 (77.50%)	
Hani	1 (0.50%)	4 (2.00%)	5 (2.50%)	
Hui	2 (1.00%)	1 (0.50%)	3 (1.50%)	
Jingpo	1 (0.50%)	1 (0.50%)	2 (1.00%)	
Lisu	2 (1.00%)	1 (0.50%)	3 (1.50%)	
Miao	4 (2.00%)	1 (0.50%)	5 (2.50%)	
Naxi	0 (0.0e+0%)	1 (0.50%)	1 (0.50%)	
Wa	1 (0.50%)	0 (0.0e+0%)	1 (0.50%)	
Yi	1 (0.50%)	8 (4.00%)	9 (4.50%)	
Zhuang	0 (0.0e+0%)	3 (1.50%)	3 (1.50%)	

Data are presented as number (percentage) for categorical variables. *P*-values were derived from the chi-square test or Fisher’s exact test for categorical variables, the independent samples *t*-test for normally distributed continuous variables, and the Mann–Whitney *U* test for non-normally distributed continuous variables.

EBV, Epstein–Barr virus.

### Clinical manifestations

As shown in **Table 2**, neurological symptoms (e.g., seizures, psychiatric/behavioral abnormalities, headache) were significantly more frequent in the EBV-positive group (*n* = 43, 66.2%) than in the EBV-negative group (*n* = 61, 45.2%; *P* = 0.007). The incidence of serous cavity effusion (pleural, pericardial, or ascitic) was also significantly higher in the EBV-positive group (*n* = 39, 60.0%) compared to the EBV-negative group (*n* = 59, 43.7%; *P* = 0.035).

**Table 2:** Differences in clinical symptoms between cSLE patients with and without EBV infection.

Variable	Positive (*n* = 65)	Negative (*n* = 135)	*P*-value
Fever	41 (63.1%)	76 (56.3%)	0.4439
Rash	44 (67.7%)	88 (65.2%)	0.7526
Oral ulcer	10 (15.4%)	25 (18.5%)	0.6927
Alopecia	5 (7.7%)	13 (9.6%)	0.7948
Arthritis	20 (30.8%)	51 (37.8%)	0.349
**Serositis**	**39 (60%)**	**59 (43.7%)**	**0.0351**
Superficial lymphadenopathy	19 (29.2%)	30 (22.2%)	0.2961
Hepatomegaly	12 (18.5%)	31 (23%)	0.5821
Splenomegaly	**11 (16.9%)**	17 (12.6%)	0.5142
**Neurological symptoms**	**43 (66.2%)**	**61 (45.2%)**	**0.0065**

Data are presented as number (percentage). *P*-values were derived from the chi-square test or Fisher’s exact test, as appropriate.

EBV, Epstein–Barr virus.The bolded parts show the results of variables with statistical differences (P < 0.05).

**Figure 1:**
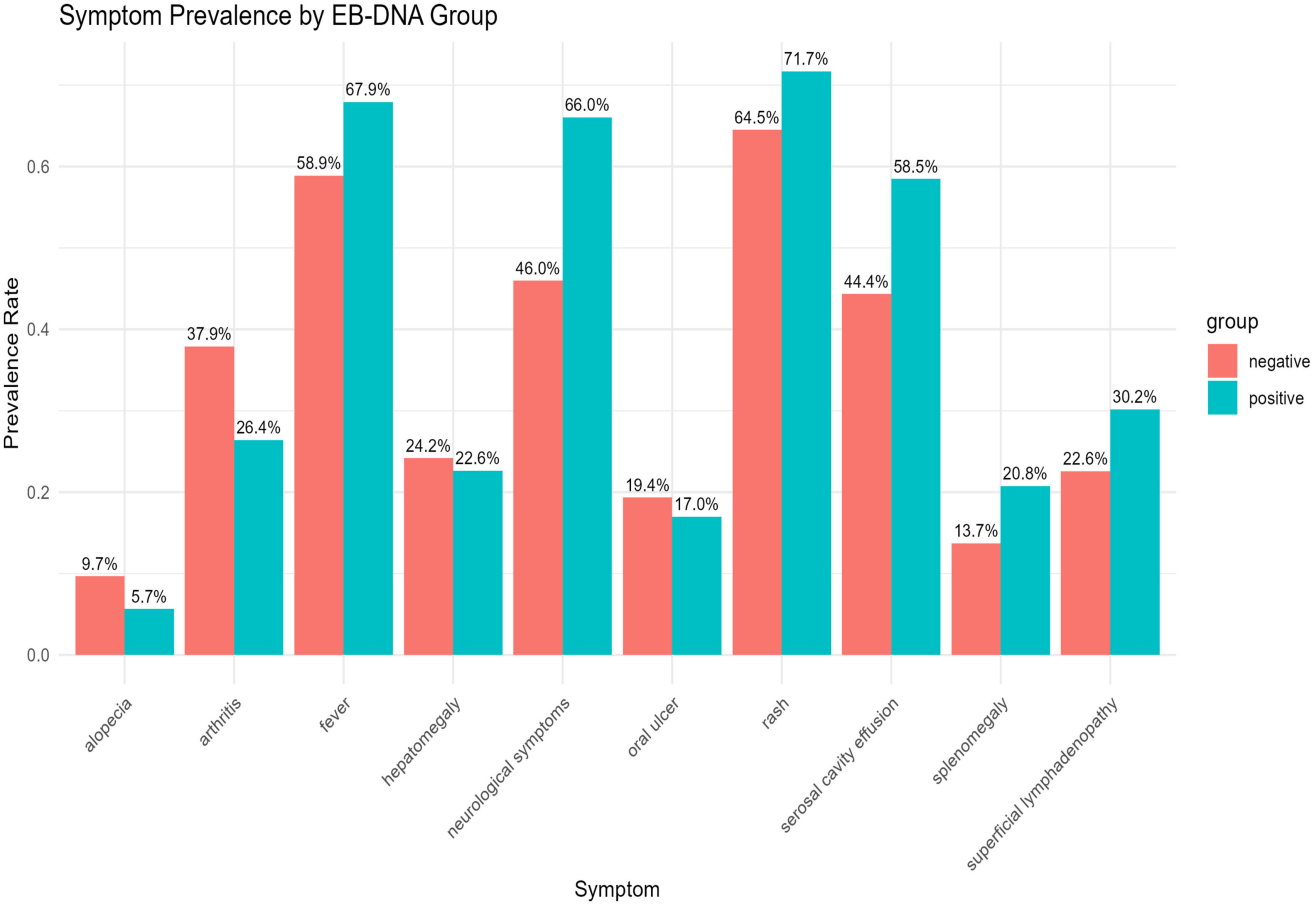
Distribution of clinical symptoms in children with SLE, stratified by EBV infection status. This bar chart presents a comparative analysis of the prevalence of various clinical symptoms between children with SLE who tested positive for Epstein–Barr virus (EBV) infection (blue bars) and those who tested negative (red bars). Statistical analysis revealed that the proportion of patients with neurological symptoms was significantly higher in the EBV-positive group (43 cases, 66.2%) compared to the EBV-negative group (61 cases, 45.2%; *P* = 0.007). Additionally, the incidence of serous cavity effusion was also significantly greater in the EBV-positive group [39 cases (60.0%)] than in the EBV-negative group [59 cases (43.7%); *P* = 0.035].

In contrast, the prevalence of other characteristic SLE manifestations—including fever (63.1% vs. 56.3%, *P* = 0.444), malar rash (67.7% vs. 65.2%, *P* = 0.753), oral ulcers (15.4% vs. 18.5%, *P* = 0.693), alopecia (7.7% vs. 9.6%, *P* = 0.795), and arthritis (30.8% vs. 37.8%, *P* = 0.349)—showed no significant differences between groups. Rates of hepatomegaly, splenomegaly, and lymphadenopathy were also comparable (all *P* > 0.05) (see **Figure 1**).

### Laboratory parameters

Comparative laboratory data are presented in **Supplementary Table 1**. Immunologically, complement C3 levels were significantly lower in the EBV-positive group (median 0.29 g/L, IQR 0.18–0.49) than in the EBV-negative group (median 0.36 g/L, IQR 0.25–0.58; *P* = 0.032). Complement C4 levels were also lower in the EBV-positive group (median 0.03 g/L, IQR 0.02–0.06 vs. median 0.04 g/L, IQR 0.03–0.08; *P* = 0.009), suggesting more pronounced complement consumption associated with EBV infection.

**Figure 2:**
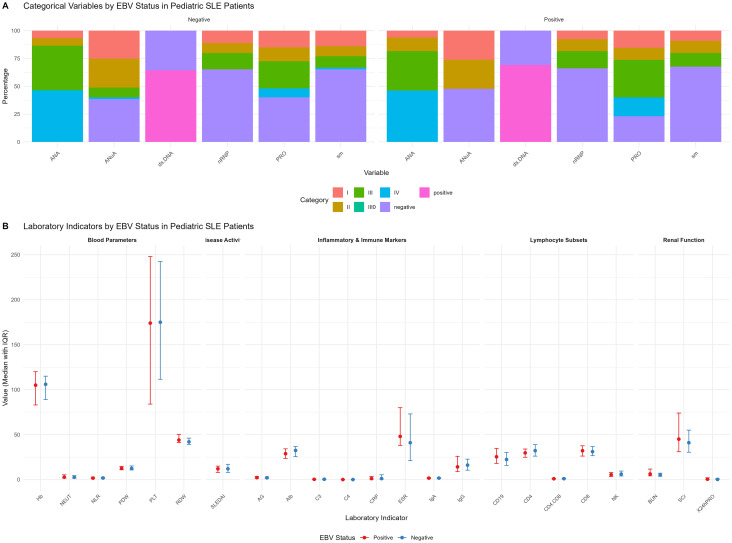
Distribution of laboratory characteristics in children with SLE, stratified by EBV infection status. **(A)** Stacked bar chart of categorical variable distribution by EBV status: This chart illustrates the positive rates of autoantibodies, including antinuclear antibody (ANA) and anti-nucleosome antibody (AnuA), among children in the EBV-positive (red) and EBV-negative (blue) groups. **(B)** Box plots of continuous variable comparison by EBV status: These box plots display the distributions of key laboratory parameters and disease activity scores—including white blood cell count (WBC), lymphocyte count (Lym), complement C3, complement C4, erythrocyte sedimentation rate (ESR), C-reactive protein (CRP), and Systemic Lupus Erythematosus Disease Activity Index 2000 (SLEDAI-2K)—comparing the EBV-positive (red) and EBV-negative (blue) groups. The box represents the interquartile range (IQR), the line inside the box indicates the median, and the whiskers show the data range (excluding outliers). Statistical analysis indicated that children in the EBV-positive group had significantly lower levels of complement C3, complement C4, and albumin, but a significantly higher level of blood urea nitrogen (BUN), compared to the EBV-negative group. No significant differences were observed in the other laboratory parameters examined.

Regarding renal function, blood urea nitrogen (BUN) was higher in the EBV-positive group (median 6.27 mmol/L, IQR 4.13–11.71) than in the EBV-negative group (median 5.10 mmol/L, IQR 3.72–7.16; *P* = 0.014), while serum albumin was lower (median 28.8 g/L, IQR 23.5–34.2 vs. median 32.4 g/L, IQR 25.5–36.8; *P* = 0.025). However, 24-h urine protein (0.42 vs. 0.19 g, *P* = 0.093) and serum creatinine levels did not differ significantly. No significant differences were observed in complete blood counts, inflammatory markers (ESR, CRP), autoantibody profiles (including anti-dsDNA, anti-Sm), or lymphocyte subsets (CD4^+^, CD8^+^, NK cells; all *P* > 0.05) (see **Figure 2**).

### Disease complications and activity

**Figure 3:**
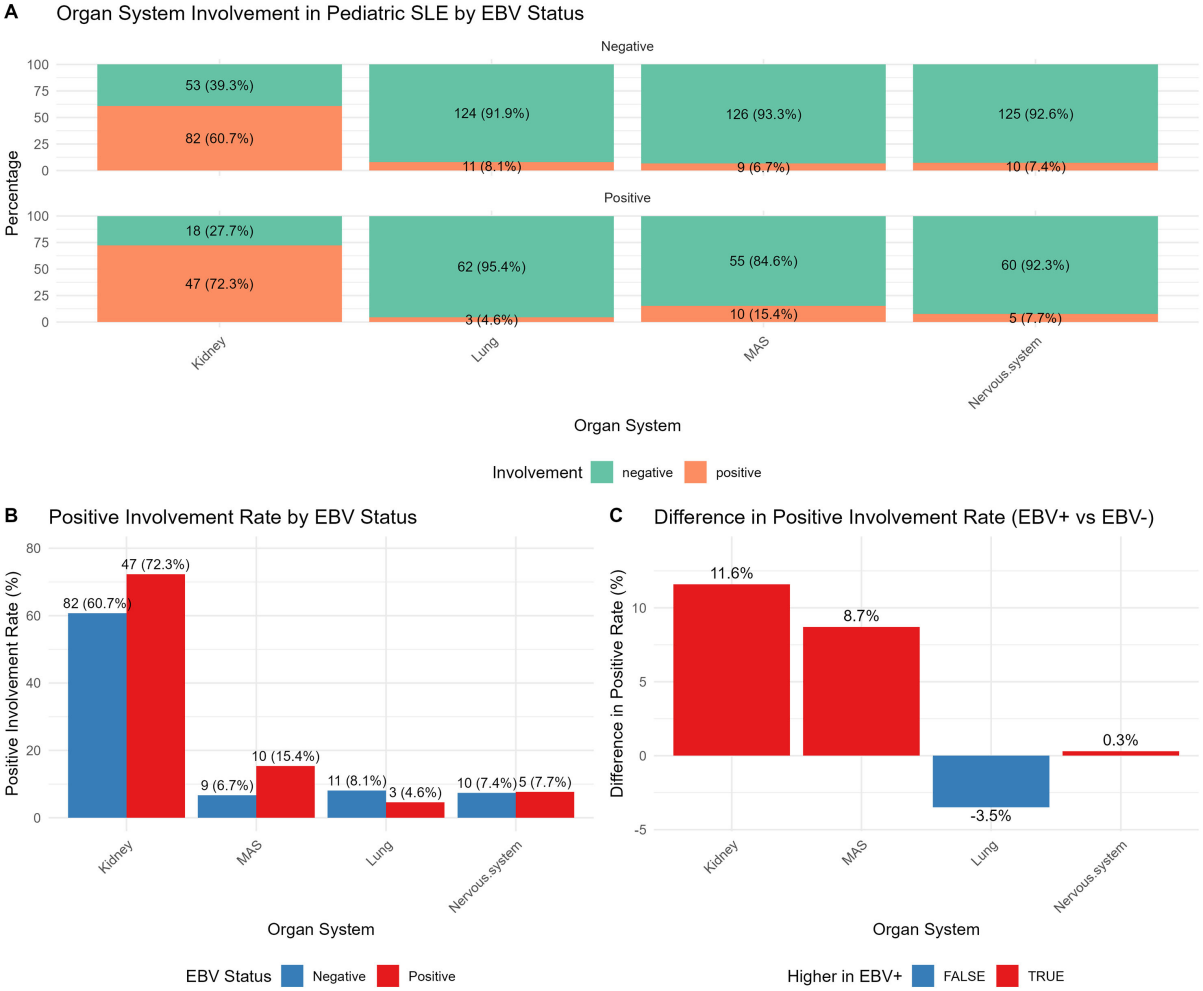
**(A–C)** Distribution of complication profiles in children with SLE, stratified by EBV infection status. This figure compares the prevalence of complications between children with SLE who are EBV-positive and those who are EBV-negative. Statistical analysis reveals a significantly higher incidence of macrophage activation syndrome (MAS) in the EBV-positive group compared to the EBV-negative group.

The incidence of MAS was significantly higher in the EBV-positive group (10/65, 15.4%) than in the EBV-negative group (9/135, 6.7%; *P* = 0.049), as shown in **Table 3**. No significant differences were found between the groups regarding the prevalence of formally diagnosed NPSLE (based on ACR criteria,7.7% vs. 7.4%, *P* = 1.000), LN (72.3% vs. 60.7%, *P* = 0.109), lupus pneumonitis (4.6% vs. 8.1%, *P* = 0.555), or the overall SLEDAI-2K score (median 12, IQR 8–15 for EBV-positive vs. median 12, IQR 8–17 for EBV-negative; *P* = 0.299), despite a higher frequency of general neurological symptoms in the EBV-positive group, indicating no association between EBV infection and overall cSLE disease activity (**Figure 3**).

**Table 3:** Distribution of complications and disease activity in pediatric SLE patients, stratified by EBV infection status.

Variable	Positive (*n* = 65)	Negative (*n* = 135)	*P*-value
Kidney	**43 (66.2%)**	**61 (45.2%)**	0.1093
Nervous system	**43 (66.2%)**	**61 (45.2%)**	1
**MAS**	**43 (66.2%)**	**61 (45.2%)**	**0.0489**
Lung	**43 (66.2%)**	**61 (45.2%)**	0.5552
SLEDAI	12 (8, 15)	12 (8, 17)	0.299

Data are presented as number (percentage). Disease activity was assessed using the Systemic Lupus Erythematosus Disease Activity Index 2000 (SLEDAI-2K) and is presented as median (interquartile range). The *P*-value for SLEDAI-2K was derived from the Mann–Whitney *U* test; *P*-values for complications were derived from the chi-square test or Fisher’s exact test.

MAS, macrophage activation syndrome; LN, lupus nephritis; NPSLE, neuropsychiatric systemic lupus erythematosus.The bolded parts show the results of variables with statistical differences (P < 0.05).

### Multivariate logistic regression analysis of MAS risk factors

Univariate analysis (**Table 4**) revealed that patients with MAS had significantly lower platelet counts (101.95 ± 65.97 vs. 186.53 ± 105.61 × 10^9^/L, *P* < 0.001), lower complement C3 levels (0.35 ± 0.35 vs. 0.43 ± 0.28 g/L, *P* = 0.04), and higher CD8^+^ T-cell percentages (37.10% ± 8.66% vs. 31.89% ± 10.74%, *P* = 0.004) compared to those without MAS. The proportions with EBV infection (52.6% vs. 30.4%, *P* = 0.049) and anti-Sm antibody positivity (47.4% vs. 32.6%, *P* = 0.046) were also significantly higher in the MAS group.

**Table 4:** Comparison of laboratory profiles between pediatric SLE patients with and without macrophage activation syndrome (MAS).

Variable	Negative (*n* = 181)	Positive (*n* = 19)	*P*-value
**EB_positive**	**55 (30.4%)**	**10 (52.6%)**	**0.049**
PRO_positive	47 (26.0%)	5 (26.3%)	1
ANA_positive	181(100%)	0 (0.0%)	1
ds.DNA_positive	118 (65.2%)	14 (73.7%)	0.457
**Sm_positive**	**59 (32.6%)**	**9 (47.4%)**	**0.046**
ANuA_positive	105 (58.0%)	12 (63.2%)	0.193
nRNP_positive	60 (33.1%)	9 (47.4%)	0.181
NEUT	3.65 ± 2.87	3.03 ± 2.99	0.104
NLR	2.39 ± 2.07	2.63 ± 3.95	0.379
Hb	104.36 ± 22.90	96.68 ± 21.70	0.130
RDW	44.32 ± 7.99	48.05 ± 9.80	0.082
**PLT**	**186.53 ± 105.61**	**101.95 ± 65.97**	**<0.001**
PDW	12.85 ± 2.86	14.04 ± 3.13	0.13
CRP	6.98 ± 18.46	19.80 ± 33.16	0.369
Alb	30.37 ± 7.77	28.17 ± 7.97	0.291
AG	2.43 ± 1.59	3.05 ± 1.87	0.117
**C3**	**0.43 ± 0.28**	**0.35 ± 0.35**	**0.04**
C4	0.07 ± 0.08	0.10 ± 0.10	0.246
BUN	6.97 ± 5.12	8.31 ± 6.93	0.532
SCr	52.24 ± 45.52	60.38 ± 36.37	0.149
IgG	17.09 ± 9.67	14.65 ± 6.66	0.443
IgA	1.73 ± 0.87	1.81 ± 0.83	0.804
ESR	51.45 ± 32.94	45.79 ± 29.43	0.536
24hPRO	0.92 ± 1.45	0.85 ± 1.76	0.7
CD19	25.55 ± 12.40	19.86 ± 9.27	0.704
CD4	31.54 ± 9.03	30.70 ± 5.68	0.061
**CD8**	**31.89 ± 10.74**	**37.10 ± 8.66**	**0.004**
NK	7.05 ± 4.89	7.11 ± 3.78	0.641

Data are presented as mean ± standard deviation or number (percentage), as appropriate. *P*-values were derived from the independent samples *t*-test, chi-square test, or Fisher’s exact test.

MAS, macrophage activation syndrome; PLT, platelet count.The bolded parts show the results of variables with statistical differences (P < 0.05).

Variables with *P <*0.1 in the univariate analysis (EBV infection, anti-Sm positivity, platelet count, complement C3, CD8^+^ T-cell %, RDW) were included in a multivariate binary logistic regression model. Multivariate analysis identified EBV infection as an independent risk factor for MAS (OR = 2.90, 95% CI: 1.01–8.69, *P* = 0.0497). Platelet count was an independent protective factor (OR = 0.25, 95% CI: 0.07–0.75, *P* = 0.0218). Anti-Sm positivity (OR = 3.43, 95% CI: 0.96–11.79, *P* = 0.0504) and higher CD8^+^ T-cell % (OR = 2.79, 95% CI: 0.97–9.33, *P* = 0.0703) showed strong trends toward being risk factors. Complement C3 lost independent significance after adjustment (*P* = 0.63) (see **Table 5**).

**Table 5:** Multivariate logistic regression analysis of independent risk factors for macrophage activation syndrome.

Variable	OR	Lower_CI	Upper_CI	*P*-value
**PLT**	**0.25**	**0.07**	**0.75**	**0.0218**
**EB**	**2.9**	**1.01**	**8.69**	**0.0497**
Sm	3.43	0.96	11.79	0.0504
CD8	2.79	0.97	9.33	0.0703
C3	0.76	0.24	2.24	0.63

The multivariate model was adjusted for variables with *P <*0.1 in the univariate analysis, including Epstein–Barr virus (EBV) infection, anti-Sm antibody positivity, platelet count, complement C3 level, CD8^+^ T-cell percentage, and red cell distribution width (RDW). Data are presented as odds ratio (OR) with 95% confidence interval (CI).

MAS, macrophage activation syndrome.The bolded parts show the results of variables with statistical differences (P < 0.05).

In summary, multivariate analysis confirmed EBV infection as an independent risk factor for MAS in cSLE, while higher platelet count was a protective factor.

## Discussion

This study is the first to investigate the association between EBV infection and clinical features in a substantial cohort of children with cSLE from Southwest China. Our key findings indicate that EBV infection is independently associated with a significantly increased risk of MAS in cSLE patients. Furthermore, it is linked to more pronounced complement activation and specific clinical manifestations, such as neurological symptoms and serous cavity effusion. However, EBV infection was not associated with an increased risk of LN or NPSLE, nor with the overall disease activity as measured by the SLEDAI-2K score. These findings provide new clinical insights into the disease heterogeneity of cSLE and the pathogenesis of MAS.

The most significant finding of this study is that EBV infection is an independent risk factor for MAS in cSLE patients (OR = 2.90, 95% CI: 1.01–8.69). This conclusion was confirmed by multivariate logistic regression analysis, which demonstrated that the association remained significant even after adjusting for other potential confounding factors such as platelet count and autoantibodies. This aligns with previous literature reporting EBV as a trigger for hemophagocytic lymphohistiocytosis (HLH) (10). The underlying mechanism may involve massive activation of CD8^+^ T cells and macrophages by EBV, leading to a cytokine storm characterized by excessive production of IFN-γ, TNF-α, IL-6, and IL-18 (11)—a pathophysiology that overlaps perfectly with MAS. Our data support this mechanism, as patients in the MAS group had a significantly higher percentage of CD8^+^ T cells (*P* = 0.004), and an elevated CD8^+^ T-cell percentage showed a strong trend toward being a risk factor for MAS in the multivariate analysis (*P* = 0.0703). Consequently, for cSLE patients with EBV infection, clinicians should maintain a high index of suspicion for its role as a potential “trigger” for MAS and enhance monitoring of relevant indicators (e.g., platelets, ferritin, fibrinogen) to facilitate early intervention.

Secondly, we found that EBV infection was associated with more significant complement consumption, evidenced by significantly lower levels of complement C3 and C4. Complement activation is a hallmark immunopathological feature of SLE. As a potent B-cell stimulant, EBV may exacerbate B-cell activation and proliferation through mechanisms like “molecular mimicry” or acting as a “bystander activator” (12), thereby promoting autoantibody production and immune complex formation, leading to more vigorous complement consumption. The trend toward a higher positive rate of anti-dsDNA antibodies in the EBV-positive group indirectly supports this hypothesis. Hypocomplementemia is a key marker of SLE disease activity (13), suggesting that cSLE patients with concurrent EBV infection might be in a state of more active immune dysregulation.

Regarding clinical manifestations, the incidence rates of neurological symptoms and serous cavity effusion were significantly higher in the EBV-positive group. The mechanisms of NPSLE and serositis are complex, potentially involving microvascular inflammation, vasogenic edema, and cytokine infiltration. EBV infection might promote these specific manifestations by exacerbating systemic inflammatory responses and vascular endothelial cell dysfunction (14). It is noteworthy that, despite the higher frequency of neurological symptoms, the incidence of formally diagnosed NPSLE did not differ between groups. This may imply that EBV-associated neurological symptoms are subtler or exist in a subclinical state.

Another noteworthy finding is that despite the higher risk of MAS and more pronounced complement consumption in the EBV-positive group, the incidence of LN was comparable to that in the negative group. This seems counterintuitive to the conventional expectation that higher disease activity should correlate with more organ damage. One possible explanation is that the immune response driven by EBV may lean more toward systemic inflammation and cytokine storm (favoring the MAS pathway) rather than targeted immune complex deposition in specific organs like the kidneys. Furthermore, multivariate analysis confirmed thrombocytopenia as an independent protective factor against MAS (OR = 0.25), which is consistent with the diagnostic criteria and pathophysiology of MAS and also reinforces the reliability of our research model.

This study has several limitations that should be acknowledged. First, the single-center, retrospective design may introduce selection bias, though we employed consecutive enrollment to minimize this potential. Second, our operational definition of EBV activity was based solely on DNA load quantification without simultaneous serological profiling to distinguish between primary infection and viral reactivation. While this approach is well-established for detecting active viral replication in clinical practice, the inability to differentiate between these two states represents a limitation in understanding the precise nature of EBV involvement in cSLE pathogenesis. Regarding the statistical methodology, we recognize that the relatively wide confidence intervals observed for some predictors in our multivariate logistic regression model, particularly for EBV infection (OR = 2.90, 95% CI: 1.01–8.69) and anti-Sm antibody positivity (OR = 3.43, 95% CI: 0.96–11.79), reflect the limited number of MAS events (*n* = 19) in our cohort. This limitation affects the precision of our effect estimates and is inherent in studies of rare complications. The variables included in the multivariate model were selected based on both clinical relevance and statistical considerations from univariate analyses (*P* < 0.1), following established methodological approaches. Importantly, we wish to clarify that all patients diagnosed with lupus nephritis (*n* = 129) in our cohort underwent renal biopsy with pathological confirmation according to the ISN/RPS classification criteria. This uniform diagnostic approach strengthens the validity of our LN assessments and eliminates potential heterogeneity from mixed diagnostic methods.

Despite these limitations, our study possesses notable strengths. The rigorous baseline assessment conducted prior to any immunosuppressive therapy provides a clear window into the initial relationship between EBV viremia and cSLE manifestations, free from treatment-related confounding. The clinical implications of our findings remain substantial: routine EBV DNA screening at diagnosis offers a straightforward strategy for identifying children at heightened MAS risk, enabling intensified monitoring from disease onset. This approach maintains clinical relevance regardless of infection type, as both primary infection and reactivation represent states of active viral replication capable of driving immune dysregulation. Most significantly, this work provides foundational evidence supporting future prospective studies. By establishing a strong baseline association between EBV viremia and MAS risk in a well-characterized cohort with uniformly confirmed LN diagnoses, our study supplies the necessary preliminary data to justify longitudinal investigations aimed at elucidating EBV’s temporal dynamics in cSLE and developing targeted management strategies.

## Conclusion

In conclusion, this study demonstrates that active EBV infection is independently associated with an increased risk of macrophage activation syndrome in cSLE patients and is linked to more significant complement consumption and specific clinical phenotypes. Larger prospective studies are warranted to further elucidate the causal role of EBV in the pathogenesis and progression of SLE and to explore whether EBV-targeted prevention or treatment strategies could yield clinical benefits for this high-risk pediatric population.

## Data Availability

The raw data supporting the conclusions of this article will be made available by the authors, without undue reservation.
