# Supplementary material for: Clinical features and comorbidities of Epstein–Barr virus infection in childhood-onset systemic lupus erythematosus with a focus on macrophage activation syndrome: a cross-sectional study of 200 patients
Source: Front Immunol. 2026 Jan 26;17:1714490. doi: 10.3389/fimmu.2026.1714490 (PMC12883786; doi:10.3389/fimmu.2026.1714490)
Supplement: Supplementary file 1 [file Table1.docx]

Supplementary Table 1. Comparison of Laboratory Parameters Between EBV-Positive and EBV-Negative Pediatric SLE Patients

| **Variable** | **Category** | **Positive(n=65)** | **Negative(n=135)** | **P_Value** |
| --- | --- | --- | --- | --- |
| PRO | I | 10 (15.4%) | 20 (14.8%) | 0.0793 |
|  | II | 7 (10.8%) | 17 (12.6%) |  |
|  | III | 22 (33.8%) | 33 (24.4%) |  |
|  | IV | 11 (16.9%) | 11 (8.1%) |  |
|  | negative | 15 (23.1%) | 54 (40%) |  |
| ANA | I | 4 (6.2%) | 9 (6.7%) | 0.7069 |
|  | II | 8 (12.3%) | 9 (6.7%) |  |
|  | III | 23 (35.4%) | 54 (40%) |  |
|  | III0 | 0 (0%) | 1 (0.7%) |  |
|  | IV | 30 (46.2%) | 62 (45.9%) |  |
| ds.DNA | negative | 20 (30.8%) | 48 (35.6%) | 0.5282 |
|  | positive | 45 (69.2%) | 87 (64.4%) |  |
| sm | I | 6 (9.2%) | 19 (14.1%) | 0.7949 |
|  | II | 7 (10.8%) | 12 (8.9%) |  |
|  | III | 8 (12.3%) | 14 (10.4%) |  |
|  | IV | 0 (0%) | 2 (1.5%) |  |
|  | negative | 44 (67.7%) | 88 (65.2%) |  |
| ANuA | I | 17 (26.2%) | 34 (25.2%) | 0.0679 |
|  | II | 17 (26.2%) | 35 (25.9%) |  |
|  | III | 0 (0%) | 12 (8.9%) |  |
|  | IV | 0 (0%) | 2 (1.5%) |  |
|  | negative | 31 (47.7%) | 52 (38.5%) |  |
| nRNP | I | 5 (7.7%) | 15 (11.1%) | 0.8808 |
|  | II | 7 (10.8%) | 12 (8.9%) |  |
|  | III | 10 (15.4%) | 20 (14.8%) |  |
|  | negative | 43 (66.2%) | 88 (65.2%) |  |
| NEUT | Continuous variable | 2.72 (1.69, 5.28) | 2.63 (1.62, 4.42) | 0.5364 |
| LYM | Continuous variable | 1.45 (1.01, 2.11) | 1.5 (1.06, 2.2) | 0.8215 |
| NLR | Continuous variable | 1.63 (1.13, 2.84) | 1.76 (1.08, 2.78) | 0.7444 |
| Hb | Continuous variable | 105 (83, 120) | 106 (89, 115) | 0.5651 |
| RDW | Continuous variable | 44 (41.1, 50.3) | 42 (39.1, 46.15) | 0.0506 |
| PLT | Continuous variable | 174 (84, 248) | 175 (111.5, 242.5) | 0.5459 |
| RPR | Continuous variable | 0.26 (0.19, 0.53) | 0.25 (0.17, 0.42) | 0.2665 |
| PDW | Continuous variable | 12.5 (10.9, 14.7) | 12.4 (10.7, 15.15) | 0.9397 |
| CRP | Continuous variable | 1.13 (0.5, 3.49) | 1.13 (0.5, 5.39) | 0.6593 |
| **Alb** | **Continuous variable** | **28.8 (23.5, 34.2)** | **32.4 (25.5, 36.8)** | **0.0249** |
| AG | Continuous variable | 2.2 (1.49, 3.55) | 2.03 (1.42, 2.95) | 0.2115 |
| **C3** | **Continuous variable** | **0.29 (0.18, 0.49)** | **0.36 (0.25, 0.58)** | **0.0317** |
| **C4** | **Continuous variable** | **0.03 (0.02, 0.06)** | **0.04 (0.03, 0.08)** | **0.0091** |
| **BUN** | **Continuous variable** | **6.27 (4.13, 11.71)** | **5.1 (3.72, 7.16)** | **0.0136** |
| SCr | Continuous variable | 45 (31, 74) | 41 (30.4, 55) | 0.1235 |
| IgG | Continuous variable | 14.13 (8.92, 25.9) | 16.1 (10.49, 22.76) | 0.5714 |
| IgA | Continuous variable | 1.64 (1.17, 2.18) | 1.65 (1.1, 2.2) | 0.9844 |
| ESR | Continuous variable | 48 (38, 80) | 41 (21, 73) | 0.0842 |
| 24hPRO | Continuous variable | 0.42 (0.05, 2.05) | 0.19 (0.04, 0.96) | 0.0934 |
| CD19 | Continuous variable | 25.36 (18.01, 34.69) | 22.34 (15.85, 30.16) | 0.117 |
| CD4 | Continuous variable | 29.7 (24.81, 34) | 32.16 (26.1, 39.03) | 0.1364 |
| CD8 | Continuous variable | 32.09 (26.17, 37.62) | 31.08 (26.66, 36.8) | 0.3951 |
| CD4.CD8 | Continuous variable | 0.96 (0.71, 1.21) | 1.03 (0.74, 1.46) | 0.137 |
| NK | Continuous variable | 5.44 (3.48, 7.88) | 6.14 (3.98, 9.51) | 0.114 |

Note:Data for normally distributed parameters are presented as mean ± standard deviation; non-normally distributed parameters are presented as median (interquartile range). P-values were derived from the independent samples t-test (for normally distributed data) or the Mann-Whitney U test (for non-normally distributed data). EBV, Epstein-Barr virus; WBC, white blood cell count; Lym#, lymphocyte count; ESR, erythrocyte sedimentation rate; CRP, C-reactive protein; SLEDAI-2K, Systemic Lupus Erythematosus Disease Activity Index 2000.
